# Draft Genome Sequences of Six Type Strains of the Genus Streptacidiphilus

**DOI:** 10.1128/genomeA.01387-14

**Published:** 2015-01-08

**Authors:** Hisayuki Komaki, Natsuko Ichikawa, Akio Oguchi, Moriyuki Hamada, Tomohiko Tamura, Nobuyuki Fujita

**Affiliations:** a Biological Resource Center, National Institute of Technology and Evaluation (NBRC), Chiba, Japan; b NBRC, Tokyo, Japan

## Abstract

Members of the genus Streptacidiphilus are acidophilic actinomycetes with streptomycete-like features. Here, we report the draft genome sequences of the type strains of Streptacidiphilus albus, Streptacidiphilus anmyonensis, Streptacidiphilus carbonis, Streptacidiphilus jiangxiensis, Streptacidiphilus melanogenes, and Streptacidiphilus neutrinimicus. These genome sequences will serve as valuable references for understanding their taxonomic relationships, genetic characteristics, and potentials for industry.

## GENOME ANNOUNCEMENT

The genus Streptacidiphilus is composed of acidophilic actinomycetes with streptomycete-like features, and it belongs to the family Streptomycetaceae (1). At present, this genus contains 10 species with validly published names. On the basis of 16S rRNA gene sequence data, these species are closely related to members of the genera Kitasatospora and Streptomyces (2), which are famous as promising sources for secondary metabolites. Members of the genus Streptacidiphilus have a major role in the turnover of organic matter in acidic habitats (3, 4) and are also reported to be potential producers of antifungal compounds (5) and acid-stable enzymes (6). Despite the increasing number of Streptomyces and Kitasatospora genome sequencing projects, only the Streptacidiphilus rugosus AM-16^T^ genome sequence (GenBank accession no. JQMJ00000000.1) had been available in the genus Streptacidiphilus, and the genomes of the other nine species had not been reported when we started this study. Since the Biological Resource Center, National Institute of Technology and Evaluation (NBRC) possesses six type strains of Streptacidiphilus species whose genomes were not sequenced, we performed whole-genome shotgun sequencing of these strains.

Genomic DNAs of Streptacidiphilus albus NBRC 100918^T^ and Streptacidiphilus jiangxiensis NBRC 100920^T^ were prepared from cultured mycelium using a Qiagen EZ1 tissue kit and an EZ1 advanced instrument (Qiagen) and sequenced by a combined strategy of shotgun sequencing with GS FLX+ (Roche) and paired-end sequencing with MiSeq (Illumina), while those of Streptacidiphilus anmyonensis NBRC 103185^T^, Streptacidiphilus carbonis NBRC 100919^T^, Streptacidiphilus melanogenes NBRC 103184^T^, and Streptacidiphilus neutrinimicus NBRC 100921^T^ were from liquid-dried cells in ampules provided from the NBRC culture collection and were sequenced by paired-end sequencing with MiSeq. These reads were assembled using the Newbler version 2.6 software. The results of the sequencing are summarized in Table 1.

**TABLE 1:** Summary of genome sequencing in the present study

Organism	Sequencing method	Reads (Mb)	Fold coverage	No. of scaffolds	Genome size (bp)	G+C content (%)	Accession no.
S. albus NBRC 100918^T^	MiSeq, GS FLX+	541	54	155	9,677,773	71.7	BBPL00000000
S. anmyonensis NBRC 103185^T^	MiSeq	637	68	153	9,386,419	71.7	BBPQ00000000
S. carbonis NBRC 100919^T^	MiSeq	560	66	139	8,457,280	71.0	BBPM00000000
S. jiangxiensis NBRC 100920^T^	MiSeq, GS FLX+	526	54	96	9,528,734	71.8	BBPN00000000
S. melanogenes NBRC 103184^T^	MiSeq	517	59	107	8,771,919	71.6	BBPP00000000
S. neutrinimicus NBRC 100921^T^	MiSeq	532	63	184	8,418,219	71.4	BBPO00000000

More recently, the genome sequences of S. albus JL83^T^ (GenBank accession no. JQML00000000.1), “Streptacidiphilus jeojiense” NRRL B-24555 (GenBank accession no. JOEH00000000.1), and Streptacidiphilus oryzae TH49^T^ (GenBank accession no. JQMQ00000000.1) were released to the public. Here, we are publishing the draft genome sequences of the six type strains. However, the genome sequences of two valid species, Streptacidiphilus durhamensis and Streptacidiphilus hamsterleyensis, are still unavailable.

The genome sequences reported here will serve as valuable references for studying secondary metabolite genes and also facilitate studies to elucidate the taxonomic relationships, genetic characteristics, and potentials for industry, such as enzyme production of the genus Streptacidiphilus.

### Nucleotide sequence accession numbers.

The draft genome sequences of S. albus NBRC 100918^T^, S. anmyonensis NBRC 103185^T^, S. carbonis NBRC 100919^T^, S. jiangxiensis NBRC 100920^T^, S. melanogenes NBRC 103184^T^ and S. neutrinimicus NBRC 100921^T^ have been deposited in DDBJ/ENA/GenBank databases under the accession numbers BBPL00000000, BBPQ00000000, BBPM00000000, BBPN00000000, BBPP00000000, and BBPO00000000, respectively. The versions described in this paper are the first versions, BBPL01000000, BBPQ01000000, BBPM01000000, BBPN01000000, BBPP01000000, and BBPO01000000, respectively.
